# A triphenylethylene nonsteroidal SERM attenuates cervical cancer growth

**DOI:** 10.1038/s41598-019-46680-0

**Published:** 2019-07-29

**Authors:** Neeraj Chauhan, Diane M. Maher, Murali M. Yallapu, Bilal B. Hafeez, Man M. Singh, Subhash C. Chauhan, Meena Jaggi

**Affiliations:** 1 Department of Pharmaceutical Sciences, University of Tennessee Health Science Center, 38163 Memphis, TN USA; 2 Sanford Research Center, USD, 57104 Sioux Falls, SD USA; 3 Saraswati Dental College, Lucknow, Uttar Pradesh India; 4 Department of Immunology and Microbiology, School of Medicine, University of Texas Rio Grande Valley, 78504 McAllen, TX USA

**Keywords:** Chemotherapy, Cervical cancer

## Abstract

Selective estrogen receptor modulator drug molecules of triphenylethylene family have gained considerable attention as anti-cancer agents. Despite recent advances in screening and development of HPV vaccines, cervical cancer remains one of the deadliest malignancies as advanced stage metastatic disease is mostly untreatable, thus warrants newer therapeutic strategies. Ormeloxifene (ORM) is a well-known SERM of triphenylethylene family that has been approved for human use, thus represents an ideal molecule for repurposing. In this study, we for the first time have demonstrated the anti-cancerous properties of ormeloxifene in cervical cancer. Ormeloxifene efficiently attenuated tumorigenic and metastatic properties of cervical cancer cells via arresting cell cycle at G1-S transition, inducing apoptosis, decreasing PI3K and Akt phosphorylation, mitochondrial membrane potential, and modulating G1-S transition related proteins (p21, cyclin E and Cdk2). Moreover, ORM repressed the expression of HPV E6/ E7 oncoproteins and restored the expression of their downstream target tumor suppressor proteins (p53, Rb and PTPN 13). As a result, ormeloxifene induces radio-sensitization in cervical cancer cells and caused potent tumor growth inhibition in orthotopic mouse model. Taken together, ormeloxifene represents an alternative therapeutic modality for cervical cancer which may have rapid clinical translation as it is already proven safe for human use.

## Introduction

Cervical cancer stands at the fourth rank among most common and deadly malignancies in women globally. In the year of 2018, there are 13,240 new cases and 4,170 death cases of cervical cancer estimated in the Unites States^1^. Incidence rates remain higher in less developed countries (15.7%), mainly in Eastern (42.7%), Middle (30.6%) and South Africa (31.5%), than more developed countries (9.9%)^2^.

Cervical cancer is primarily associated with persistent infection of high risk Human Papillomavirus (HPV)^3^. HPVs are circular, double stranded DNA viruses that consist of approximately 8000 base pairs^4^ and are the most common type of sexually transmitted virus infection^5^. There are more than 100 identified human papillomaviruses which are divided into two categories – High Risk and Low Risk^6^. Persistent infection of any of these 13 High risk HPV’s (HVP 16, 18, 31, 33, 35, 39, 45, 51, 52, 56, 59 and 66) can result in the progression of cervical cancer^7^. HPV 16 and 18 are the most shared (transmitted) type of HPVs and account for about 70% of invasive cervical cancer cases^8^.

Malignant cervical cancer cells specifically express two viral oncoproteins HPV E6 and E7^9^. HPV E6 and E7 are known to cause genomic alterations resulting in gain or loss of function of genes in various cancers including cervical cancer^10,11^. E6 and E7 disturb the functions of p53 (protein 53) and Rb (Retinoblastoma protein), two tumor suppressor proteins. Both proteins regulate cell cycle progression and excessive cell proliferation. The E6 binds to p53 and degrades it by proteosomal degradation^12^. Whereas the E7 oncoprotein binds to Rb, causing its degradation and leading to the induction of cell growth and gene promoting DNA synthesis^13^.

PI3K-Akt is a cell survival pathway which is responsible of many cellular functions such as cell proliferation, cell survival and cell cycle progression in variety of cancers^14^ and is activated by HPV E6 and E7^15^. Akt becomes phosphorylated at serine and threonine residuals by the overexpression of PI3K^16^ and thus, gets activated for the further initiation of apoptosis cascade^17^. Human cervical cancer tissues samples express increased levels of pAkt^18^, therefore, PI3K-Akt signaling pathway has gained much attention as potential therapeutic targets for cervical cancer^19^.

Though HPV is the most common factor of cervical cancer, yet alone it is not sufficient to develop cervical cancer and requires several chromosomal changes and genetic damages/alterations, including viral DNA integration itself and mutations mainly in TP53 and Rb^20–22^, along with other risk factors such as smoking, multiple sexual partners, early sexual debut and low socioeconomic status^23^. Genetic aberrations play a pivotal role in the progression of cervical cancer^20^. Two significant and recurrent HPV 16/18 viral DNA integrations occur specifically at 8q24 and 12q15 chromosomal locations^24–27^. Some other frequently occurring chromosomal alterations that are not even associated to p53 mutation are 3p14–22, 4p16, 5p15, 6p21–22, 11q23, 17p13.3, 18q12–22 and 19q13^28,29^. Among females, cigarette smoking has two times higher risk of having cervical cancer than those who do not smoke^30^. A well-known cigarette smoke carcinogen, BaP (Banzo[a]pyrene) increases the expression levels of HPV oncoproteins E6 and E7 in cervical cancer cells^31^. Epidemiology of cervical cancer suggests that having multiple sexual partners and early sexual debut increase the risk for HPV infection and development of cervical cancer among women^32,33^.

In many developed countries Pap Smear Screening (Pap test) to detect and subsequently remove pre-cancerous lesions has greatly reduced the incidences of cervical cancer^34^. HPV testing is also available to detect high risk HPVs and can be used independently or in combination with Pap test^35^. Current available treatment modalities for the management of cervical cancer are disease stage dependent; early stage patients are mainly advised surgery or radiotherapy whereas late stages of cervical cancer are tackled by radiation along with cisplatin based chemotherapy^36^.

Ormeloxifene (Centchroman), C_30_H_35_O_3_N.HCl, is a non-steroidal, non- hormonal anti- estrogen oral contraceptive for human use that is taken once per week^37^. Ormeloxifene belongs to triphenylethylene family^38^ and members of this family are known to have potent anti-cancer properties^39–43^. Ormeloxifene has been used as an anti-neoplastic agent in MCF-7 and MDA-MB-231 Estrogen Receptor (ER ± ve) Human Breast Cancer Cells (HBCCs)^44^. Additionally, in a phase II clinical trial of advanced stage breast cancer, ormeloxifene has shown profound anti-cancer efficacy with overall 38.7% response rate in female patients and 6 month median duration of response^37^.

In this present study, we assessed ormeloxifene’s anti-cancerous properties in cervical cancer cells and mouse model. We report that ormeloxifene effectively inhibits the cellular proliferation, growth and motility, induces apoptosis through mitochondrial intrinsic pathway and arrests cell cycle progression in cervical cancer cells. Moreover, ormeloxifene significantly decreases/inhibits the oncogenic signaling of HPV E6/7 and PI3K-Akt pathway and upregulates the tumor suppressor signaling by restoring the expression levels of p53, Rb and PTPN13. Additionally, ormeloxifene radio-sensitizes cells *in vitro* and exhibits excellent anti-tumor activity in orthotopic mice model of cervical cancer. Findings from this study, collectively, suggest that ormeloxifene has great potential to become a novel therapeutic agent for the management of cervical cancer.

## Results

### Ormeloxifene treatment inhibits cellular growth and motility of various cervical cancer cells

**Figure 1:**
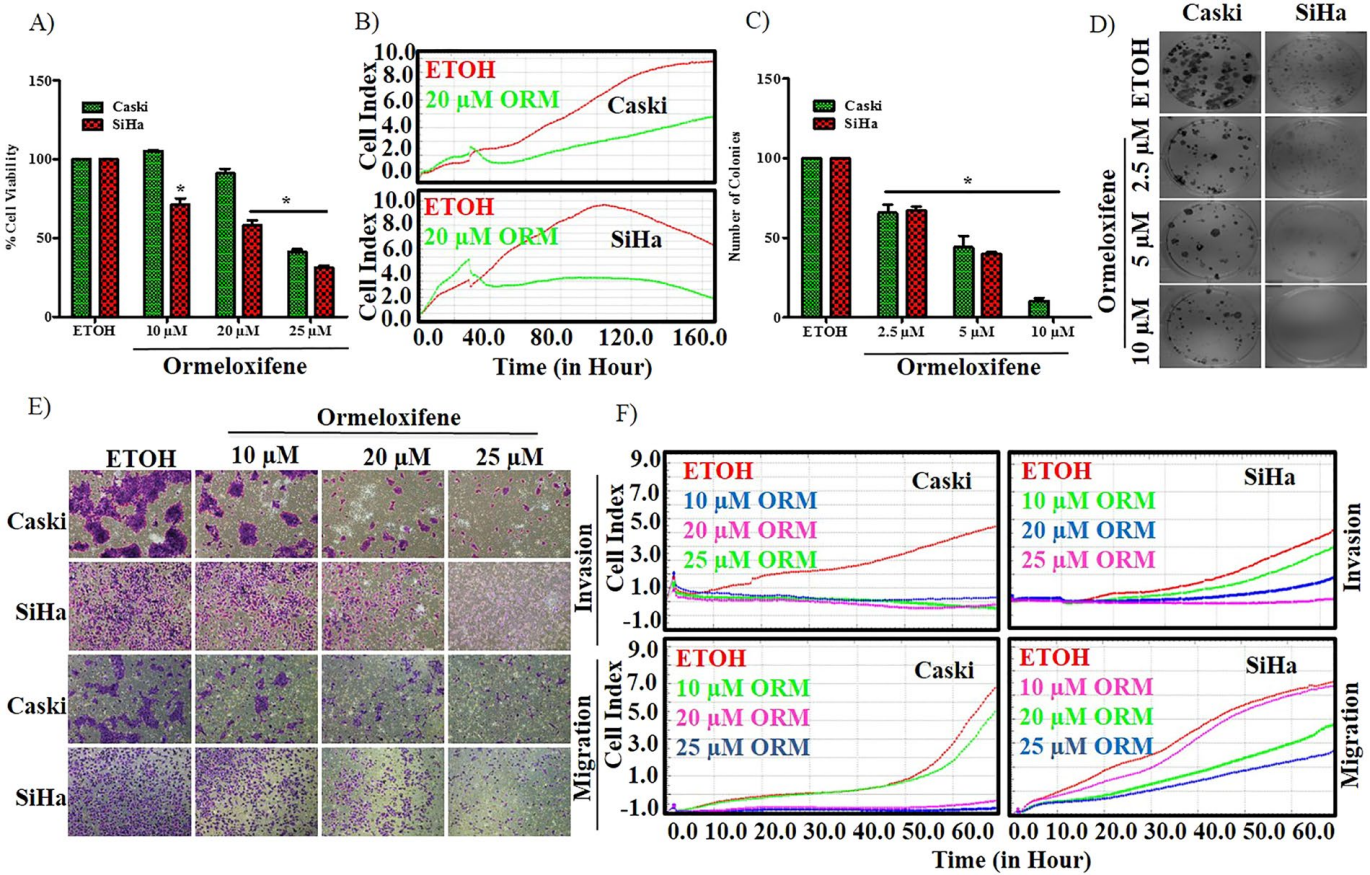
Ormeloxifene inhibits cell proliferation and motility. (**A**) Ormeloxifene decreases cellular proliferation of Caski and SiHa cells. Caski and SiHa cells were treated with ormeloxifene (10, 20, 25 µM) for 48 hours and MTS method was used to determine proliferation and absorbance was measured at 490 nm. Results were normalized to the vehicle control (ETOH). Error bars show SEM, n = 3. *p < 0.05. (**B**) Growth kinetics through xCELLigence RTCA. Caski and SiHa cell lines were treated with 20 µM ormeloxifene and growth kinetics (rate of real time proliferation) was measured. (**C,D**) Ormeloxifene inhibits clonogenic potential of cells. (**C**) Cells showed inhibited colony forming ability after 15 days of ormeloxifene treatment. Results were normalized to the ETOH control. Error bars show SEM, n = 3. *p < 0.05. (**D**) Qualitative representation of inhibited clonogenecity of cells. Images were taken at 200X. (**E**) Ormeloxifene decreased the cellular migration and invasion. Cells were treated with ormeloxifene for 24 hours and images were taken at 100X. Both cells show clear inhibition of motility and invasion confirmed by Boyden chamber method. (**F**) Motility kinetics through xCELLigence RTCA. Real time migratory and invasive properties of Caski and SiHa cells were also confirmed using xCELLigence system.

To determine the effect of ormeloxifene on cell growth of various cervical cancer cells, we performed cell proliferation (MTS) assays with Caski and SiHa (HPV positive) (Fig. 1A) and, C33A and HT3 (HPV negative) (Fig. S1A). Cells were treated with ormeloxifene at micro-molar ranges for 48 hours. All four cell lines showed a significant decrease in a dose-dependent manner and a drastic inhibitory effect was found between 20 µM and 25 µM doses. A growth kinetic experiment was also performed using xCELLigence RTCA system (Fig. 1B) to confirm ormeloxifene’s effect on cellular growth of Caski and SiHa cell lines with respect to time. Colony forming ability is an essential property of cancerous cells. Thus, we assessed colony forming assays to determine the long-term effect of ormeloxifene on cervical cancer cell lines. Ormeloxifene showed a significant effect on clonogenic potential of all tested cervical cancer cell lines (Figs. 1C,D,S1B,C) in a dose-dependent manner. We also analyzed the metastatic properties of cervical cancer cells after ormeloxifene treatment with cell migration and invasion assays using Boyden chamber migration and Boyden chamber matrigel invasion assays. Both Caski and SiHa cells showed an inhibition of migration and invasion (Fig. 1E) with an increase in ormeloxifene concentration. A real time kinetic assessment for migration and invasion was also performed using xCELLigence RTCA system (Fig. 1F) to confirm ormeloxifene’s effect on metastasis of Caski and SiHa cells, and results were consistent with the Boyden chamber assays. Furthermore, the migratory ability of cells was analyzed by using an agarose bead assay (Fig. S1D). Ormeloxifene treatment again showed an inhibition of migration in dose and time dependent manner in both cell lines.

### Ormeloxifene induces cell death through mitochondrial intrinsic pathway

**Figure 2:**
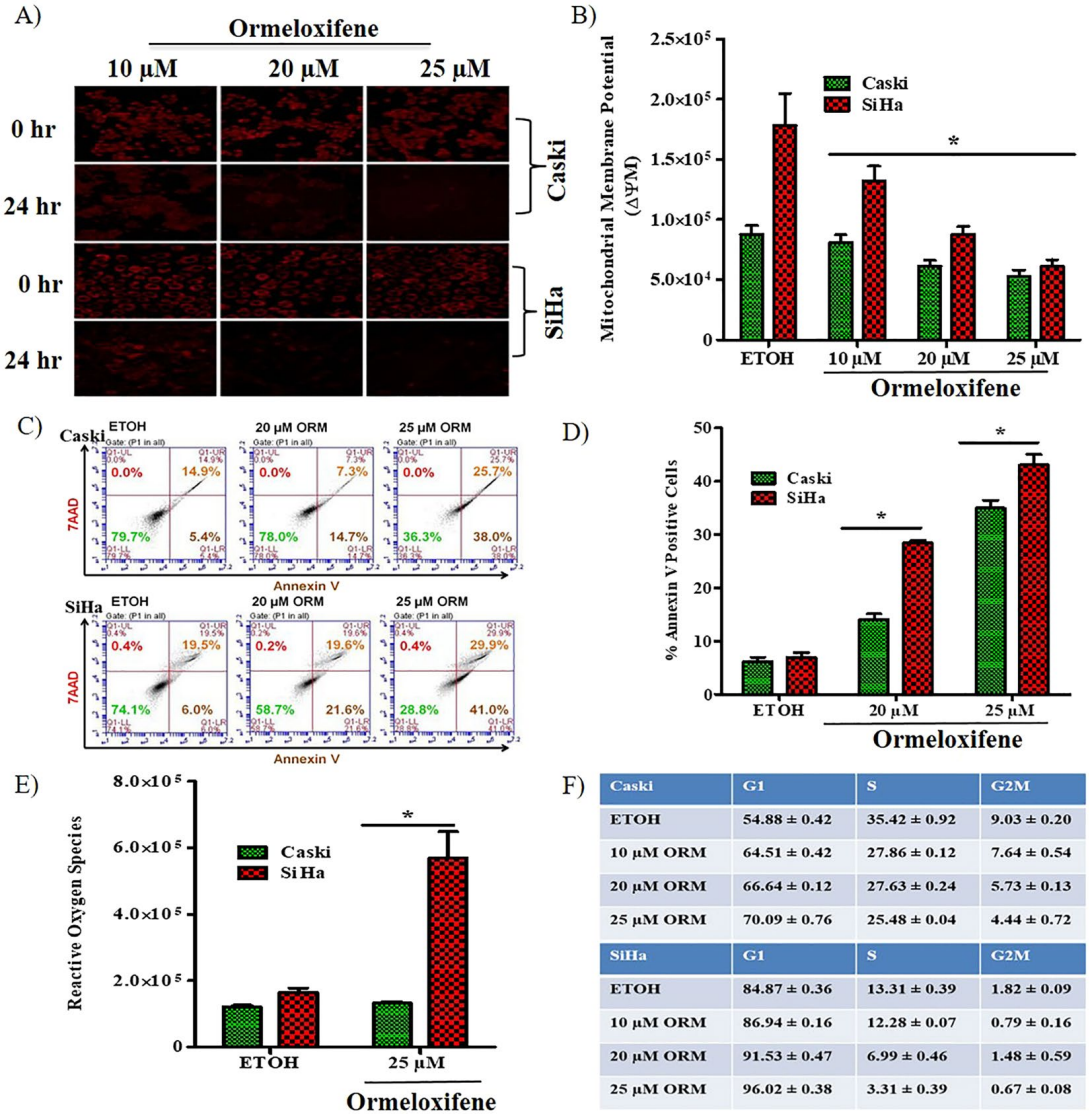
Ormeloxifene induces apoptosis and arrests cell cycle of cervical cancer cells. Ormeloxifene decreases mitochondrial membrane potential (MMP). (**A**) Cells were stained with TMRE dye for 20 mins at 37 °C and next treated with ormeloxifene for 24 hours to detect the healthy mitochondria. Representation of qualitative images showed decreased TMRE stain signifying reduced MMP, images were taken at 200X. (**B**) Flow cytometry results also showed a reduction in MMP (decreased TMRE fluorescence level). Results were normalized to the ETOH control. Error bars show SEM, n = 3. *p < 0.05. Ormeloxifene induces apoptosis. (**C**) Cells were treated with ormeloxifene for 24 hours and analyzed by flow cytometry using Annexin V and 7AAD dyes. (**D**) Graphical representation of flow cytometry data for Annexin V positive cells (early apoptosis). Error bars show SEM, n = 3. *p < 0.05. (**E**) Generation of reactive oxygen species (ROS). Cells were treated with 25 µM ormeloxifene and stained with DCFH-DA dye. Flow cytometry data represented an elevated levels of DCFH-DA dye which denotes generation of ROS. Error bars show SEM, n = 3. *p < 0.05. (**F**) Cell cycle was arrested at G1-S transition. Cells were treated with ormeloxifene for 24 hours, stained with PI dye and analyzed by flow cytometer and ModFit software for cell cycle analysis. ± shows SEM, n = 3. *p < 0.05.

Decreased mitochondrial membrane potential (MMP) is a clear sign of induction of apoptosis through the mitochondrial intrinsic pathway. We observed cells by microscopy and also performed flow cytometry using TMRE (Tetramethyl rhodamine ethyl ester, Invitrogen) stain to detect the depolarization of mitochondrial membrane in Caski and SiHa cells. Ormeloxifene significantly decreases MMP of both cell lines (Fig. 2A,B). Cells were also observed under phase contrast microscopy to visualize signs of apoptosis, such as cell membrane blebbing and shrinkage (Fig. S2A). Furthermore, we confirmed ormeloxifene’s ability to induce apoptosis by staining the cells with Annexin V-7AAD dyes. Both cell lines showed a marked increase in the percentage of Annexin V positive cell population (Fig. 2C,D) at 20 and 25 µM concentrations after 24 hours of treatment. Under stress conditions, such as drug treatment or radiation, tumor cells are known to generate reactive oxygen species (ROS) due to the oxidative stress. Production of reactive oxygen species is one of the key signals that leads to apoptosis. Therefore, we used DCFH-DA (dichlorodihydrofluorescein diacetate) stain to detect the amount of reactive oxygen species generation after ormeloxifene treatment in Caski and SiHa cell lines. DCFH-DA is commonly used to detect intracellular production of H_2_O_2_. After 24 hours of ormeloxifene treatment at 25 µM concentration both cell lines showed elevated levels of H_2_O_2_ generation when compared to vehicle control ETOH as confirmed by higher intensity levels of DCFH-DA stain (Fig. 2E). We further validated ormeloxifene’s ability to block cell cycle progression by performing the cell cycle arrest assay to determine the cell population at different phases of cell cycle. Caski and SiHa cell lines were treated with 10, 20 and 25 µM of ormeloxifene for 24 hours and stained with Propidium Iodide (PI). Cells showed a blockage of cell cycle progression at G1-S transition (Figs. 2F,S2B).

### Ormeloxifene modulates the expression of cell cycle regulatory proteins and PI3K-Akt pathway

**Figure 3:**
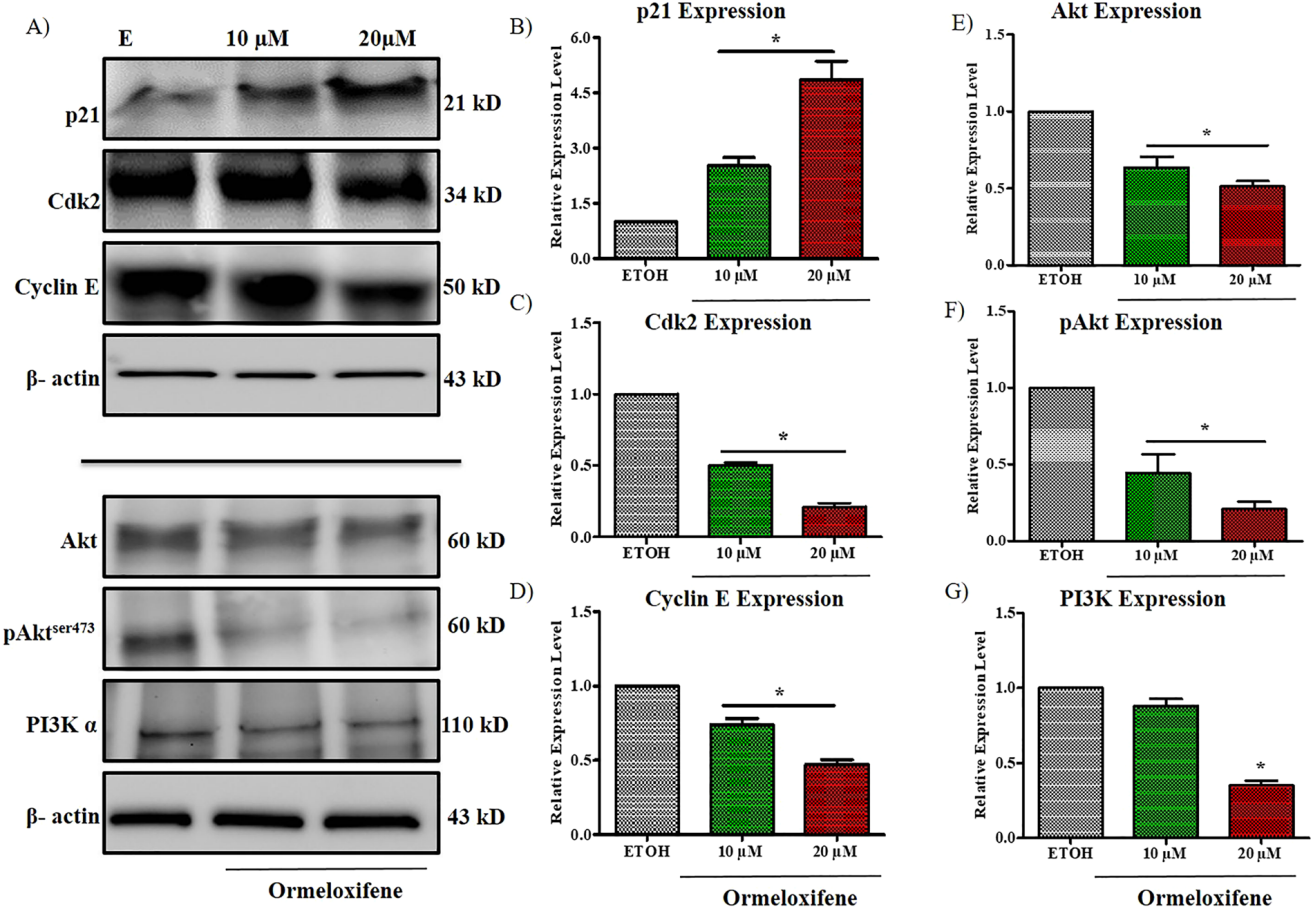
Ormeloxifene modulates the expression of cell cycle regulatory proteins and decreases PI3K/Akt pathway. (**A**) Immunoblots for protein expression. Caski cells were treated with ormeloxifene for 24 hours and immunoblots were preformed to detect p21, CdK2, Cyclin E, Akt, pAkt and PI3K proteins. β -actin was used as a loading control. All full-length blots are presented in Supplementary Information. Band quantifications of (**B**) p21, (**C**) Cdk2, (**D**) Cyclin E, (**E**) Akt, (**F**) pAkt, and (**G**) PI3K. Band quantitation was done by using GelQuant software. Band intensity was normalized to β -actin and scaled to the ETOH control. Bars = Relative Expression Level, Error bars show SEM, n = 3. *p < 0.05.

We further confirmed the cell cycle arrest at G1-S transition with immunoblotting by probing for two transition related molecules Cyclin E and its dependent kinase; cyclin dependent kinase 2 (Cdk2) and also for p21 which is a known repressor of this transition phase. Ormeloxifene significantly decreased the expression of Cyclin E and Cdk2 and increased the expression of p21 at 20 µM concentration when compared to the vehicle control ETOH (Fig. 3A top panel). PI3K-Akt cell survival oncogenic pathway is overly expressed in cervical cancer cells. The pathway is required for cell cycle progression from G1 phase to S phase. Inhibition of PI3K-Akt pathway is an important tool for developing the treatment strategies for cervical cancer. We utilized immunoblotting assay to evaluate the inhibitory effects of ormeloxifene on PI3K-Akt pathway. Ormeloxifene slightly decreased the total Akt expression but pAkt, which is the phosphorylated and active form of Akt, and PI3K were decreased markedly (Fig. 3A bottom panel). We also quantified the band intensities of these immunoblots (Fig. 3B through 3G).

### Ormeloxifene decreases the expression of HPV E6 and E7

**Figure 4:**
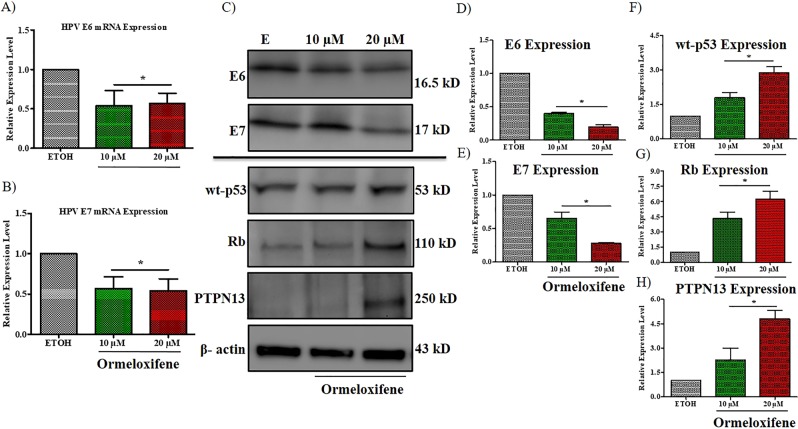
Effects of ormeloxifene on HPV infection. Ormeloxifene decreases (**A**) HPV E6 and (**B**) E7 mRNAs. Caski cells were treated with ormeloxifene for 6 hours and mRNA levels of HPV E6 and E7 were determined by quantitative PCR. Expression levels were normalized to a house keeping gene (β-2-Microgloblin) and scaled to the vehicle control (ETOH). Bars = Relative Expression Level, Error bars show SEM, n = 3. *p < 0.05. (**C)** Ormeloxifene inhibits HPV E6 and E7 proteins and upregulates tumor suppressor proteins. Caski cells were treated with ormeloxifene for 24 hours and western blots were preformed to detect HPV E6 and E7, wt-p53, Rb and PTPN13 proteins. β -actin was used as a loading control. All full-length blots are presented in Supplementary Information. Band quantifications of (**D**) E6, (**E**) E7, (**F**) wt-p53, (**G**) Rb and (**H**) PTPN13. Band quantitation was done by using GelQuant software. Band intensity was normalized to β -actin and scaled to the ETOH control. Bars = Relative Expression Level, Error bars show SEM, n = 3. *p < 0.05.

Oncogenes E6 and E7 are responsible for continuous cell division which promotes the development of cervical cancer. An effect on the expression of human papillomavirus E6 and E7 is an important consideration for cervical cancer treatment. Quantitative RT-PCR was performed to determine the expression of HPV oncogenes E6 and E7 in Caski cells. Ormeloxifene significantly inhibited the expression of E6 and E7 after 6 hours of ormeloxifene treatment (10 and 20 µM doses) (Fig. 4A,B). The indicated treatment was compared with vehicle control ETOH. We also assessed the downregulation of oncogenic signaling at the translational level by performing immunoblotting. Both oncoproteins showed a marked reduction in the expression level compared to the vehicle control ETOH (Fig. 4C top panel). The oncogenic properties of HPV E6 and E7 are increased when E6 and E7 bind to the tumor suppressor proteins p53, Rb and PTPN13. These tumor suppressor proteins p53, Rb and PTPN13 are not mutated in HPV positive cervical cancer cells but are inactivated (or low in expression/function)^45,46^. Ormeloxifene’s effect on these tumor suppressing molecules was determined by the immunoblotting assays. We treated Caski cervical cancer cells with 10 and 20 µM concentrations of ormeloxifene for 24 hours and probed for these three tumor suppressor proteins. We observed a slight but noticeable increase in the expression of wild type p53 but Rb and PTPN13 proteins showed a marked increase in the expression after ormeloxifene treatment at 20 µM dose (Fig. 4C bottom panel), band intensities for these blots were also measured (Fig. 4D through 4H).

### Ormeloxifene treatment induces radio-sensitization in cervical cancer cells

**Figure 5:**
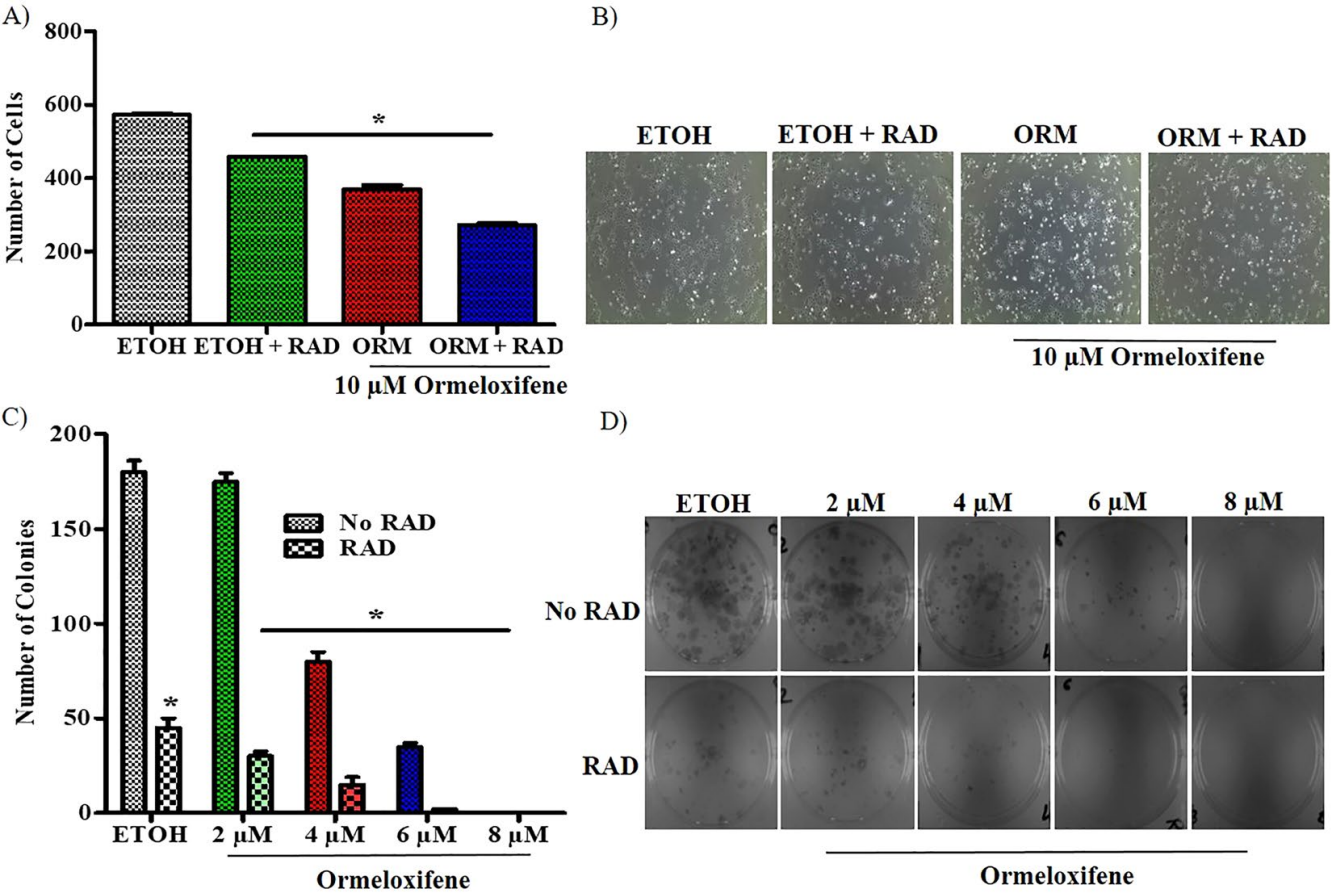
Ormeloxifene sensitizes Caski cells to radiation. Ormeloxifene radio-sensitizes cells and decreases cell proliferation. (**A**) Caski cells were pretreated with 10 µM ormeloxifene for 6 hours and next exposed to 4Gy radiation. Live cells were counted using a coulter counter. Error bars show SEM, n = 3. *p < 0.05. (**B)** Representative images of ormeloxifene and radiation treated cells. Images were taken at 100X. Ormeloxifene radio-sensitizes cells and decreases colony forming ability. (**C)** Caski cells were pretreated with 2, 4, 6 and 8 µM ormeloxifene for 6 hours and next exposed to 4Gy radiation. After 14 days, cells with combination treatment showed fewer colonies than compared to ormeloxifene and radiation alone. Error bars show SEM, n = 3. *p < 0.05. (**D)** Images represent inhibited clonogenic potential. Images were taken at 200X.

We performed short-term and long-term studies to analyze ormeloxifene’s effect on radio-sensitization in Caski cells. We observed that after 24 hours of radiation, number of live cells significantly decreased with 6 hours pretreatment of ormeloxifene when compared with radiation alone and ormeloxifene alone treatment groups (Fig. 5A,B). These results were consistent with long-term treatment of radiation. Pretreatment of ormeloxifene significantly sensitized Caski cells to radiation as confirmed by decreased number of colonies when compared to radiation alone and ormeloxifene alone treated groups (Fig. 5C,D).

### Ormeloxifene effectively inhibits cervical cancer tumorigenesis in orthotopic mice model

**Figure 6:**
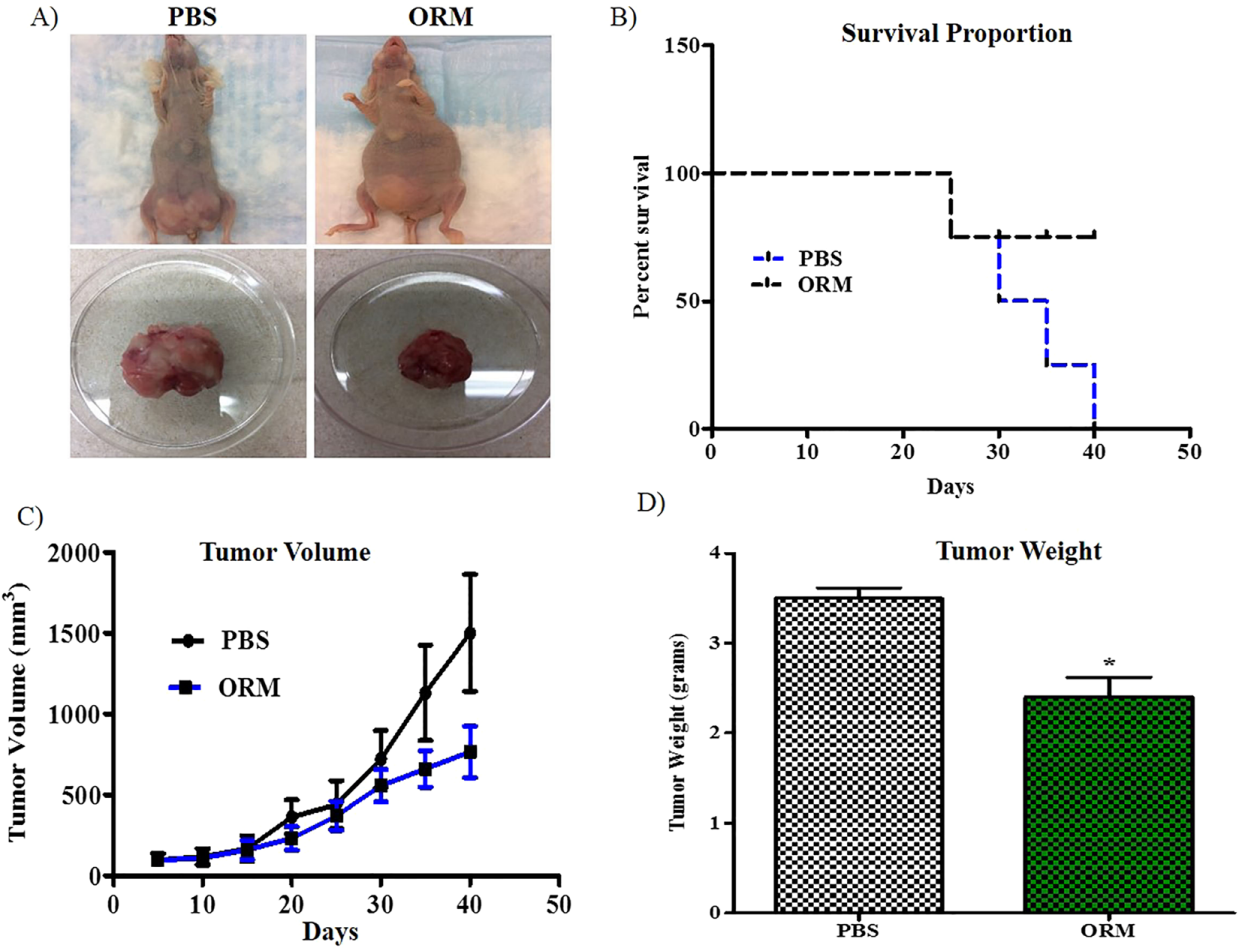
Ormeloxifene inhibits tumor growth in cervical cancer orthotopic mice model. Orthotopic mice model was generated by injecting Caski cells direct into the cervix of female nude mice. (**A**) Images represent mice from different treatment groups. (**B**) Ormeloxifene increased overall life expectancy and mice survival than compared to its vehicle control PBS. (**C**,**D**) Ormeloxifene also showed a marked reduction in tumor volume and weight than compared to PBS. Error bars show SEM, n = 6. *p < 0.05.

It was important to test ormeloxifene *in vivo* after observing its anti-cancerous properties in *in vitro* models. In order to evaluate anti-tumorous efficacy of ormeloxifene, we generated an orthotopic cervical cancer mouse model using female nude mice. For this mouse model development, we used HPV positive Caski cervical cancer cells and injected directly into the cervix of these female nude mice. 6 mice were taken in each treatment group (PBS and ORM; 2 treatment groups). Mice started developing tumors within 15–20 days, and once the tumor size was approximately 100 mm^3^, ormeloxifene and its vehicle control PBS were administered by intra-peritoneal injections. The study was terminated when the control group tumor size reached around 1000 mm^3^. At termination, mice were euthanized, and tumors were dissected from both groups (Fig. 6A). It was observed that ormeloxifene increased the overall mice survival rate (Fig. 6B) when compared to its vehicle control PBS group. During the entire study tumor size/volume was measured. Ormeloxifene significantly decreased the tumor volume when compared to its vehicle control PBS (Fig. 6C). We also measured the tumor weight after dissection and results suggested that ormeloxifene reduced the tumor weight markedly when compared to its vehicle control PBS (Fig. 6D).

## Discussion

Despite of having various preventative modalities including vaccines and screening^34^, cervical cancer is a deadly malignancy of women^3^. Advanced stages of cervical cancer remain untreatable because of many modulations at the cellular and molecular levels, therefore, cells become more invasive and show resistance towards chemo and radiotherapy^36,47^. Thus, newer therapeutic means are highly desirable that provide protection against metastasis and chemo/radio-resistance. Currently, repurposing of the molecules which are already in use has become a new attraction^48^. Considering the potential of repurposing a drug that is already in use, we investigated the therapeutic potential of ormeloxifene in this study. Ormeloxifene has been in use for its birth control proposes for more than two decades in India^49^. Ormeloxifene targets high proliferative decidule cells in the endometrium and has been reported to be safe^50,51^. Recently, studies from other groups and our lab have exhibited that ormeloxifene has anti-cancerous activities against breast, head and neck, ovarian, pancreatic and leukemia cancers^52^.

Uncontrolled cell proliferation/growth and inability to have programmed cell death are the major causes of cancer development^53^. In this study, we show that ormeloxifene decreases cellular proliferation of four different cell lines, HPV positive and negative, with a short-term (48 hours) treatment of ormeloxifene as confirmed by MTS assay (Figs. 1A,S1A). Results from xCELLigence real time growth kinetic assay are also consistent with MTS data findings (Fig. 1B). Ormeloxifene also inhibits the clonogenic potential of all four cell lines with a long-term treatment of about 14/15 days (Figs. 1C,D,S1B,S1C). These results are consistent with previous work showing ormeloxifene’s anti-proliferative potential^40,42^. When cells go under drug treatment, they exhibit apoptosis like signs, ormeloxifene treatment alters the morphology of Caski and SiHa cells and shows the apoptosis like signs such as membrane blebbing, cell shrinkage and rounding of shape (Fig. S2A)^54^. Apoptosis is a major event that occurs in response to many anti-cancer drugs^54^. Apoptosis consists of two different pathways, namely extrinsic and intrinsic. Intrinsic apoptotic death cascade involves the depolarization of mitochondrial membrane as a first event of the cascade that further leads to the activation of caspases and results in PARP cleavage^55^. Ormeloxifene treatment for 24 hours affects the mitochondrial membrane depolarization as confirmed by decreased mitochondrial membrane potential assessed by TMRE staining through fluorescent microscopy and flow cytometer (Fig. 2A,B). Annexin V-7AAD staining is another method to detect the apoptotic cell population. Ormeloxifene treatment for 24 hours results in increased apoptotic population of Caski and SiHa cell lines (Fig. 2C,D), also ormeloxifene activates ROS generation in these cells (Fig. 2E), which indicates that ormeloxifene has the potential to become anti-proliferative/growth molecule for cervical cancer.

Excessive cell cycle progression is a key property of cancerous cells and controlled by many activated cyclins and their dependent kinases^56,57^. Ormeloxifene treatment plays an inhibitory role in cell cycle progression of Caski and SiHa cells. It arrests cell cycle in both cell lines at G1-S transition (Figs. 2F,S2B) and decreases the expression of Cyclin E and its dependent kinase CDk2 (Fig. 3A top). Cyclin E and Cdk2 help cells to progress from G1 phase to S phase^56^. PI3K-Akt pathway plays an important role in cell growth and cell cycle progression^58^. In accordance with previous findings^40^, we show that ormeloxifene downregulates the PI3K-Akt pathway in cervical cancer cells as shown by decreased expression levels of PI3K, Akt and pAkt (Fig. 3A bottom). Ormeloxifene further increases the downstream target of this pathway, p21 (Fig. 3A top), which is also a known inhibitor of cell cycle progression. Cancer cells show enhanced motility and invasion which help cancer cells migrate from one organ to another and this is how cancer spreads^59^. Molecules that inhibit migratory and invasive property of cancer cells are strong candidates for having anti-cancerous properties. We utilized various techniques including agarose bead assay (Fig. S1D), Boyden chamber migration and invasion assays (Fig. 1E), and xCELLigence real time kinetic studies (Fig. 1F) to confirm ormeloxifene’s effect on cellular motility of Caski and SiHa, results reveal that cells become less motile and invasive with ormeloxifene treatment for 24 hours.

Persistent HPV infection is required for the progression of cervical cancer^60^, so we intended to assess ormeloxifene’s effect on HPV E6 and E7 expression. Ormeloxifene successfully inhibits the mRNA levels of HPV E6 and E7 with 6 hours of treatment (Fig. 4A,B), the downregulation also continued to the translational levels as ormeloxifene decreases the expression of HPV E6 and E7 oncoproteins with 24 hours treatment (Fig. 4C top). HPV E6 and E7 deregulate the cell cycle by inactivating tumor suppressor proteins p53, Rb and PTPN13^12,13,61^, interestingly, ormeloxifene upregulates the expression of these three proteins (Fig. 4C bottom). These findings suggest that ormeloxifene not only decreases the oncogenic signaling but also increases the tumor suppressing signaling, thus, have potent anti-HPV properties in cervical cancer cells. Additionally, we observed that ormeloxifene sensitizes cervical cancer cells to radiation (Fig. 5A–D) and shows great anti-tumor efficacy (Fig. 6A–D).

Available literature suggests that ormeloxifene has strong anti-neoplastic properties in MCF-7/ MDA MB-231 Estrogen Receptor (ER ± ve) Human Breast Cancer Cells (HBCCs)^62,63^. Moreover, ormeloxifene works excellent in combinational therapies as well with curcumin or resveratrol^64^ and with glycine soya^65^ against breast cancer; results from these studies suggest that ormeloxifene works via both ER dependent and ER independent mechanisms and also it seems to work through various other pathways such as PI3K-Akt, STAT3 and SHH^40–42^ which are responsible for proliferation and cell cycle progression. In addition, data presented in this study clearly indicates that ormeloxifene modulates HPV E6/E7 signaling and PI3K-Akt pathway in cervical cancer. Thus, collectively this information denotes that ormeloxifene is a multifunctional/multifaceted molecule and targets different signaling pathways in different types of cancers. Previously published studies show that HPV E6/E7 activates and alters the function of phosphorylated form of Akt (serine/threonine) in both Rb dependent^15^ and independent^66^ manner. Therefore, this can be interpreted that ormeloxifene driven inactivation of PI3K-Akt signaling in this study is HPV E6/E7 mediated but future experiments are warranted to determine the underline molecular mechanism for ORM’s action in cervical cancer as ormeloxifene has been reported to work through both HPV dependent and independent mechanisms.

## Conclusion

To conclude, the present study clearly shows that ormeloxifene significantly decreases cell growth, migration, invasion, arrests cell cycle and induces apoptosis through mitochondrial death cascade. Also, ormeloxifene downregulates PI3K-Akt cell survival signaling. Interestingly, ormeloxifene reduces the expression of HPV E6 and E7, and as a result of this inhibited oncogenic signaling it further restores the tumor suppressors p53, Rb and PTPN13. Moreover, it radio-sensitizes cells and improves life expectancy of tumor bearing mice. Therefore, collectively, findings from this study suggest that ormeloxifene has great potential to become a novel treatment strategy for overall cervical cancer management.

## Material and Methods

### Chemicals and antibodies

All chemicals and reagents were purchased from Sigma-Aldrich Corporation (St. Louis, MO, USA) and all other cell culture consumables were purchased from Corning life sciences (Tewksbury, MA, USA), unless otherwise mentioned. Specific antibodies of HPV 16 E6/E7 were procured from Abcam (Cambridge, MA, USA), antibodies of PI3K, Akt, pAkt, Rb, PTPN13, p21, Cyclin E, Cdk2 and β-actin were obtained from Cell Signaling Technology Inc. (Danvers, MA, USA) and p53 was purchased from Santa Cruz Biotechnology Inc. (Santa Cruz, CA, USA). Details on Ormeloxifene (ORM) was kindly provided by M.M.S.

### Cell culture

All cells (HPV positive Caski and SiHa, HPV negative C33A and HT3) were obtained from ATCC. SiHa and C33A were cultured in DMEM supplemented with 4500 mg/L glucose, 4.00 mM L- Glutamine, 10% heat inactivated FBS (Atlantic Biologicals, Lawrenceville, GA, USA), 1% (5 ml) sodium pyruvate, 1% (5 ml) nonessential amino acids and 1% (5 ml) 1X antibiotic/antimycotic (Sigma-Aldrich, St. Louis, MO, USA). Caski cells were maintained in RPMI medium containing 2.05 mM L-Glutamine, 10% heat inactivated FBS and 1% (5 ml) 1X antibiotic/antimycotic. HT3 cells were grown in McCoy’s 5A medium accommodated with L-Glutamine, 10% heat inactivated FBS and 1% (5 ml) 1X antibiotic/antimycotic. All cells were incubated at 37 °C in a humidified atmosphere of 5% CO_2_.

### Cell proliferation

We used four different cervical cancer cell lines; Caski, SiHa, C33A and HT3. All cell lines were seeded at 5000 per well in 96-well plates and allowed to attach overnight. Following day, all cervical cancer cell lines were treated with 10, 20 and 25 µM concentrations of ormeloxifene for 48 hours. After 48 hours, the CellTiter96 Aqueous One Solution (Promega, Madison, WI, USA) was added to 96-well plates (20 µL/well) for 2 hours incubation at 37 °C and absorbency was measured at 490 nm. Proliferation results were normalized to control wells treated with vehicle control ETOH. The experiment was performed in 6 replicates and repeated for three times.

### Clonogenic potential

All cervical cancer cell lines were seeded at 200/well (Caski, SiHa and C33A) and 500/well (HT3) in 6-well plates and allowed to adhere overnight. Following day, cells were treated with 2.5, 5 and 10 µM of ormeloxifene and maintained for next 15 days. On the 15^th^ day cells were washed, fixed in cold methanol and stained with hematoxylin (Thermo Fisher, MA, USA). Visible colonies (~50 cells) were counted and presented as compared to the vehicle (ETOH) control. Experiment was done in 3 replicates and repeated for 3 times.

### Boyden chamber cell migration and invasion

Cell migration assay was performed in a 96-well format HTS transwell plate from Corning. Caski and SiHa cells were serum starved for overnight and next day, plated at 30,000 cells/well in upper chambers containing 100 µL serum free medium with 10, 20 and 25 µM ormeloxifene treatments and allowed to migrate towards FBS gradient to lower chamber with 10% FBS for next 24 hours. Next, cells were fixed with 4% paraformaldehyde for 30 mins at room temperature and stained with crystal violet for next 30 mins. Plates were then washed with running water to remove any excess staining. Cells in the upper chamber were completely removed by cotton swab while wet, plates were then air-dried and migrated cells were imaged by using light microscope at 100X. For invasion assay, the same protocol was used other than cells were plated in 24-transwell format from BD Biosciences in 500 µL serum free medium and ormeloxifene treatment was given next morning.

### Agarose bead assay

For this experiment, 6-well plates were pre-coated with BSA-fibronectin. Cells (1 × 10^5^) were trypsinized and mixed into a 0.2% low melting point agarose solution. About 30 µL of cells/agarose suspension was then plated onto fibronectin/BSA coated plates to form a bead. The agarose-beaded plates were placed at 4 °C for 10 mins to set the agarose beads. Complete growth medium containing drug treatments was slowly added from the edges of the wells so that beads were not disturbed and completely covered, and plates were then placed into a cell culture incubator at 37 °C with 5% CO_2_. At 0 and 24 hours, the plates were imaged under a phase-contrast microscope at 100X.

### Real time cell proliferation, migration and invasion kinetic by xCELLigence

The xCELLigence system was used to measure the cellular events in real time as it is an electrical impedance-based method. Caski and SiHa cell lines for cell proliferation (8 × 10^3^ per chamber) and for migration/invasion (3 × 10^4^ per chamber) were seeded and exposed to ormeloxifene and vehicle control (ETOH) treatments. Chamber plates were incubated in xCELLigence instrument unit at 37 °C with 5% CO_2_ for real time cell proliferation, migration and invasion measurements.

### Morphology alteration

Both cells were plated at 1 × 10^6^ per 100 mm dish and allowed to adhere overnight. Next day, cells were treated with a range of ormeloxifene (10, 20 and 25 μM) for 24 hours. Next, images were taken by using phase contrast microscopy at 200X for apoptosis like signs including cell rounding, cell shrinkage and membrane blebbing.

### Mitochondrial membrane potential

Caski and SiHa cell lines were plated at 1 × 10^6^/plate and allowed to attach overnight. Next day, cells were incubated with 2 mL phenol red free medium containing 50 nM TMRE (Thermo Fisher, MA, USA) at 37 °C for 20 mins, washed twice with PBS and exposed to ormeloxifene treatment for next 24 hours. At the indicated time, cells were washed twice with PBS, supplied with phenol red free medium and imaged using an Olympus microscope at 200X. For 0 hour images, cells were imaged right after the drug treatment. After imaging, cells were washed with PBS, trypsinized and analyzed with Accuri C6 flow cytometer (BD Biosciences, CA, USA) in FL2 channel.

### Annexin V-7AAD staining

Caski and SiHa cell lines were plated (1 × 10^6^/plate) and allowed to attach overnight. Next morning, cells were treated with different concentrations of ormeloxifene for 24 hours, both floaters and adherent cells were collected, washed twice with PBS and stained with Annexin V and 7AAD (BD Biosciences, CA, USA) 5 µL of each/100 µL of cell suspension for 20 mins in the dark at room temperature. After the incubation, cells were analyzed with Accuri C6 flow cytometer (BD Biosciences, CA, USA) in FL2 channel.

### Cell cycle analysis

Caski and SiHa cell lines were plated in 100 mm dish at 1 × 10^6^/plate and allowed to adhere overnight. Next day, cells were exposed to ormeloxifene treatment for 24 hours, trypsinized, washed twice with PBS, fixed with 70% ice cold ETOH and saved at −20 °C until further used. Cells were stained with Propidium Iodide solution (Sigma-Aldrich, St. Louis, MO, USA) 50 µg in 1 mL Telford reagent at 1 mL for 1 × 10^6^ cells for 4 hours at 4 °C in the dark and examined by Accuri C6 (BD Biosciences, CA, USA) flow cytometer in FL2 channel. Data was further analyzed, and histograms were prepared using ModFit software.

### Reactive oxygen species (ROS)

Both cell lines were plated at 1 × 10^6^/100 mm dish and allowed to adhere overnight. Next morning, cells were exposed to 25 µM ormeloxifene for 24 hours. After 24 hours, cells were washed with PBS, trypsinized, stained with 20 µM DCFH-DA (Sigma-Aldrich, St. Louis, MO, USA) (a dye that is widely used to detect generation of ROS) in 1 mL PBS and incubated for 20 mins at 37 °C. Cells were next analyzed with Accuri C6 flow cytometer (BD Biosciences, CA, USA) in FL2 channel.

### Immunoblotting

We selected Caski as our experimental model for this and next experiments as Caski contains almost 600 copies of viral genome^67^ and thus, represents the best working model for cervical cancer. Caski (1 × 10^6^) cells were plated per 100 mm dish and allowed to attach overnight. Next day, cells were treated with different concentrations of ormeloxifene for 24 hours. After treatment, cells were collected in 2X SDS lysis buffer (Santa Cruz Biotechnologies, Santa Cruz, CA, USA), sonicated and the protein concentration was normalized using SYPRO- Orange (Molecular Probes, OR, USA). SDS-PAGE electrophoresis was done using 4–20% or 10% gels and resolved proteins were transferred onto PVDF (BioRad, Hercules, CA, USA) membrane. HPV 16 E6/E7 (Abcam, MA, USA), PI3K, Akt, pAkt, Rb, PTPN13, p21, Cyclin E and Cdk2 (Cell Signaling, MA, USA) and p53 (Santa Cruz, CA, USA) primary antibodies were used. The primary antibodies were detected by an HRP-secondary antibody (anti-mouse or anti-rabbit) followed by incubation with the Lumi-Light detection reagent (Roche, Nutley, NJ, USA) and developed/imaged by using a UVP gel documentation system (all full-length blots are presented in Supplementary Information). Densitometry analyses of western blot bands were performed by using GelQuant software. Band intensity was normalized with the β -actin loading control and the expression level was compared to the vehicle control (ETOH).

### Quantitative PCR

1 × 10^6^ Caski cells were plated per 100 mm dish and allowed to attach overnight. Next, cells were treated with ormeloxifene at 10 and 20 µM for 6 hours. After indicated time, adherent cells were collected, and RNA was extracted with the Qiagen RNeasy kit (Qiagen Inc., Valencia, CA, USA). Reverse transcription was performed with the High Capacity RNA to cDNA kit and amplified with SYBR Green PCR master mix (both from Applied Biosystems). The cDNA was amplified with primers specific for HPV16 E6, HPV16 E7 and β2-microglobulin. E6-F: **caaaccgttgtgtgatttgttaatta;** E6-R: **gctttttgtccagatgtctttgc;** E7-F: **ccggacacagcccattacaa;** E7-R: **cgaatgtctacgtgtgtgctttg;** β2M-F: **tgagtatgcctgccgtgtga;** β2M-R: **tgatgctgcttacatgtctcgat**^63^. Each reaction was performed in duplicate with a MyiQ single color real time PCR thermo cycler and analyzed with Genex (Microsoft Excel macro provided by BioRad). The results were first normalized to the endogenous control (β2-microglobulin) and then the expression level was scaled to the vehicle control (ETOH).

### Radio-sensitization

In order to analyze the sensitization effects of ormeloxifene with radiation we utilized Caski cells. For cell proliferation, cells were seeded at 200,000 cells per well of 6-well plates and allowed to adhere overnight. Next day, cells were pretreated with 10 µM ormeloxifene for 6 hours and then exposed to 4Gy dose of radiation and maintained for another 24 hours. A Biological X-ray irradiator (Radiation Source, Alpharetta, GA, USA) was used to radiate the cells. After the indicated time cells were trypsinized, washed with PBS and counted with coulter counter (Beckman coulter counter, Life Sciences, IN, USA). Experiment was done in triplicate. For clonogenic potential, cells were seeded at 200 cells per well in 6-well plates and allowed to attach. Following day, cells were pretreated with ormeloxifene at 2, 4, 6 and 8 µM for 6 hours. After 6 hours, cells were exposed to radiation at 4Gy dose. Next, cells were maintained for 12–14 days. On the day of termination, cells were washed, fixed in cold methanol and stained with hematoxylin (Thermo Fisher, MA, USA). Visible colonies (~50 cells) were counted manually. Each experiment was done in triplicates.

### *In vivo* orthotopic tumoral study

In order to study the anti-tumoral properties of ormeloxifene, we developed an orthotopic mice model for cervical cancer. 4–6 weeks-old nu/nu female mice were purchased from Jackson laboratories and maintained in a pathogen-free environment. All experimental procedures were carried out in accordance with relevant guidelines and regulations as per provided in the protocols approved by the UTHSC Institutional Animal Care and Use Committee (UTHSC IACUC). We utilized 2 treatment groups named as PBS and ORM and each group had 6 mice. Caski cells (5 × 10^6^ per mouse) were suspended in PBS and matrigel (BD Bioscience, CA, USA) at a 1:1 ratio. Cell suspension (5 × 10^6^ cells in 100 µL) was injected orthotopically (direct) to the cervix of each mouse. Mice were monitored for the tumor development and once tumor reached the size of around 100 mm^3^, we started the drug treatment. Mice were treated with ORM and its vehicle control PBS at the concentration of 100 µg/mouse (ORM dose was selected based on our previously published study^42^) intra-peritoneally (i.p.) for the systemic therapy. Tumor volumes were measured by using a digital vernier caliper and calculated using the ellipsoid formula of tumor volume (mm^3^) = π/(6 × L × W × H), and survival was noted up to day 40. Mice survival was analyzed by Kaplan Meir analysis. Mice were sacrificed during the experiment if tumor volume/weight reached more than 10% of body weight (1.5–2.0 gram) or if mice lost more than 10% of wt. of their total body weight. On day 40, mice were euthanized, and tumors were observed and dissected.

### Statistical methods

The data presented as mean ± standard error (SEM). Statistical analysis was determined by using an unpaired, two tailed student’s t-test. The results were considered significant if p < 0.05. All graphs were generated using GraphPad software.

## Supplementary information

Supplementary Information
