# Supplementary material for: A triphenylethylene nonsteroidal SERM attenuates cervical cancer growth
Source: Sci Rep. 2019 Jul 29;9:10917. doi: 10.1038/s41598-019-46680-0 (PMC6662837; doi:10.1038/s41598-019-46680-0)
Supplement: Supplementary file 1 — Supplementary Information [file 41598_2019_46680_MOESM1_ESM.pdf]

A triphenylethylene nonsteroidal SERM attenuates cervical cancer growth

Neeraj Chauhan[1,4], Diane M. Maher[2], Murali M. Yallapu[1,4], Bilal B. Hafeez[1,4], Man M. Singh[3], *Subhash C. Chauhan[1,4], *Meena Jaggi[1,4]

[1]Department of Pharmaceutical Sciences, University of Tennessee Health Science Center, Memphis, TN, USA, 38163, [2]Sanford Research Center, USD, Sioux Falls, SD, USA, 57104, [3]Saraswati Dental College, Lucknow, Uttar Pradesh, India, [4]Department of Immunology and Microbiology, School of Medicine, University of Texas Rio Grande Valley, McAllen, TX, USA, 78504

Financial Support: This work was supported by NIH UO1CA162106 and RO1CA142736. This work was also partially supported by RO1CA204552, RO1CA210192 and RO1CA206069.

*Correspondence and requests for materials should be addressed to:

***Subhash C. Chauhan, PhD**
Chair and Professor
Department of Immunology and Microbiology, School of Medicine
University of Texas Rio Grande Valley
McAllen, TX, USA, 78504
Phone: (956)-296-5000
Email: schauha1@uthsc.edu OR subhash.chauhan@utrgv.edu

***Meena Jaggi, PhD**
Professor
Department of Immunology and Microbiology, School of Medicine
University of Texas Rio Grande Valley
McAllen, TX, USA, 78504
Phone: (956)-296-1926
Email: mjaggi@uthsc.edu OR meena.jaggi@utrgv.edu

Conflict of interest: No potential conflicts of interest were disclosed.

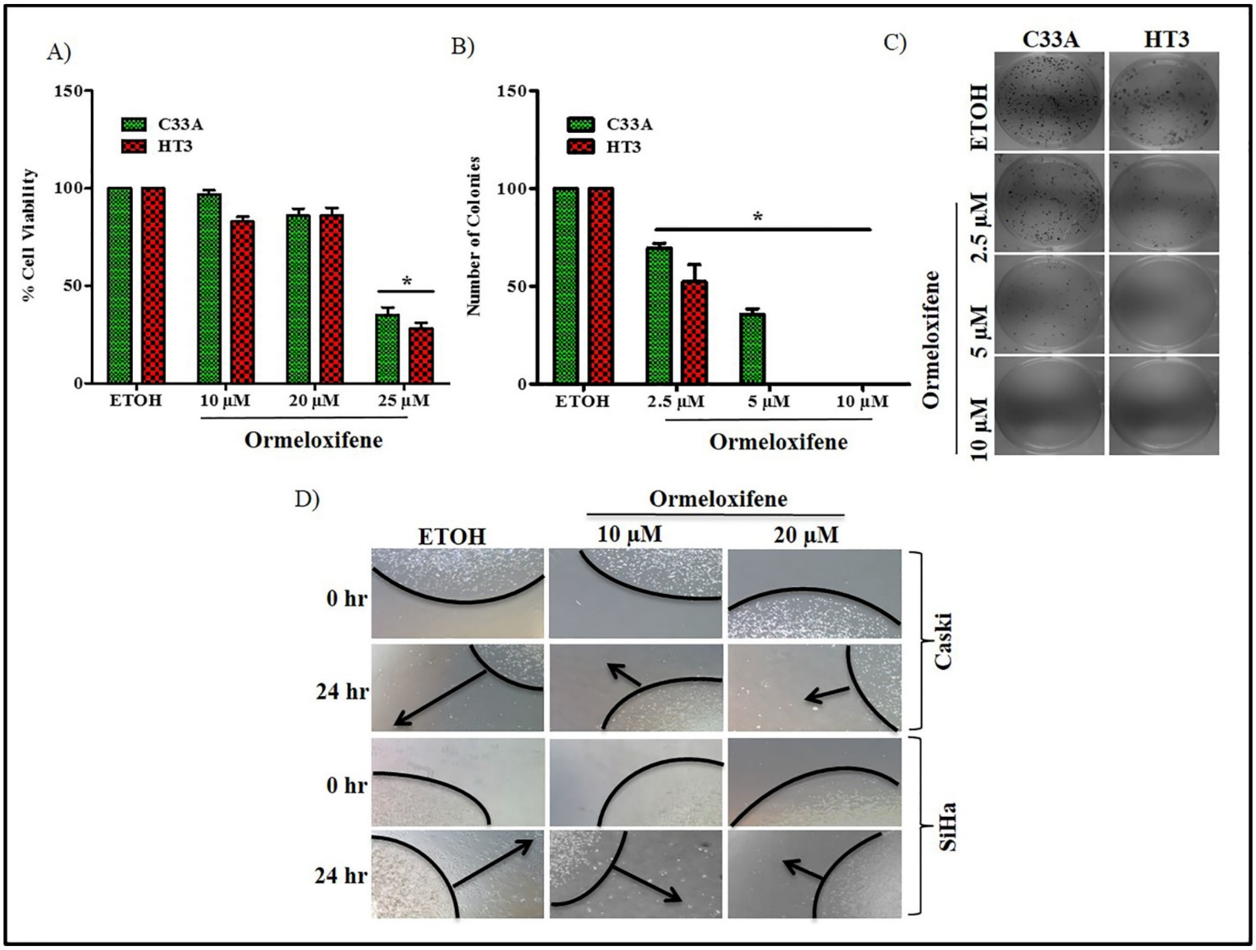

<u>Figure S1:</u> Ormeloxifene inhibits cell proliferation and motility. (A) Ormeloxifene decreases cellular proliferation of C33A and HT3 cells. C33A and HT3 cells were treated with ormeloxifene (10, 20, 25 μM) for 48 hours and MTS method was used to determine proliferation and absorbance was measured at 490 nm. Results were normalized to the vehicle control (ETOH). Error bars show SEM, n=3. *p<0.05. **(B,C) Ormeloxifene inhibits clonogenic potential of C33A and HT3 cells. (B)** Cells showed inhibited colony forming ability after 15 days of ormeloxifene treatment. Results were normalized to the ETOH control. Error bars show SEM, n=3. *p<0.05. **(C)** Qualitative representation of inhibited clonogenecity of cells. Images were taken at 200X. **(D). Agarose bead assay.** Caski and SiHa cell lines were treated with 10 and 20 μM ormeloxifene,

mixed with agarose and a bead was formed with 30 μL of this mixture. Cells were imaged at 0 and 24 hours and observed for migrated cells. Images were taken at 100X.

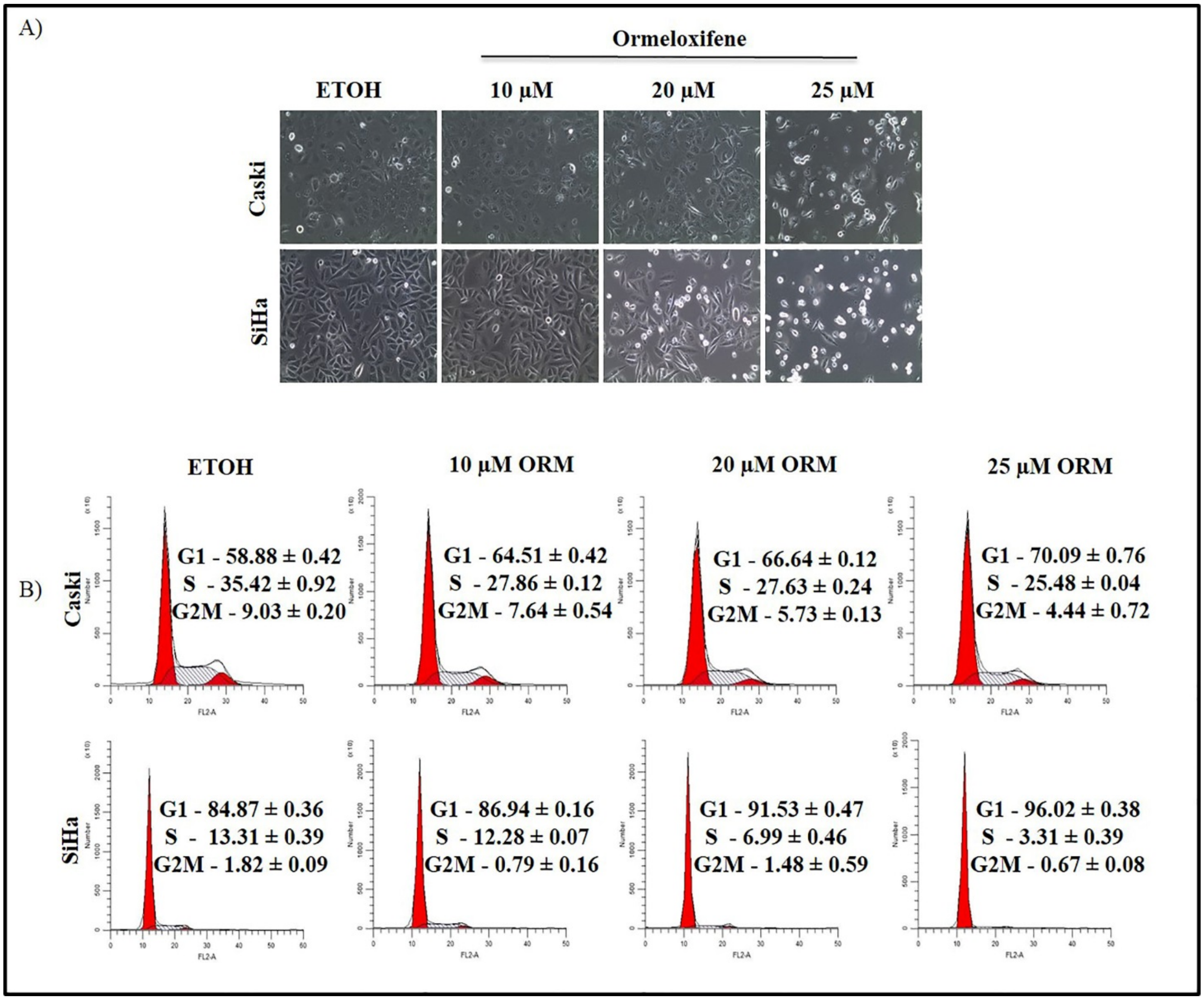

<u>**Figure S2:**</u> **Ormeloxifene induces apoptosis and arrests cell cycle of cervical cancer cells. (A) Ormeloxifene alters morphology of cells**. Caski and SiHa cells were observed and imaged under a phase contrast microscope at 200X after 48 hours of ormeloxifene treatment. Cells showed clear signs of apoptosis including blebbing and shrinkage. **(B) Cell cycle is arrested at G1-S transition.** Histograms represent percentage cells distribution at different phases of cell cycle. Data was generated using ModFit software.

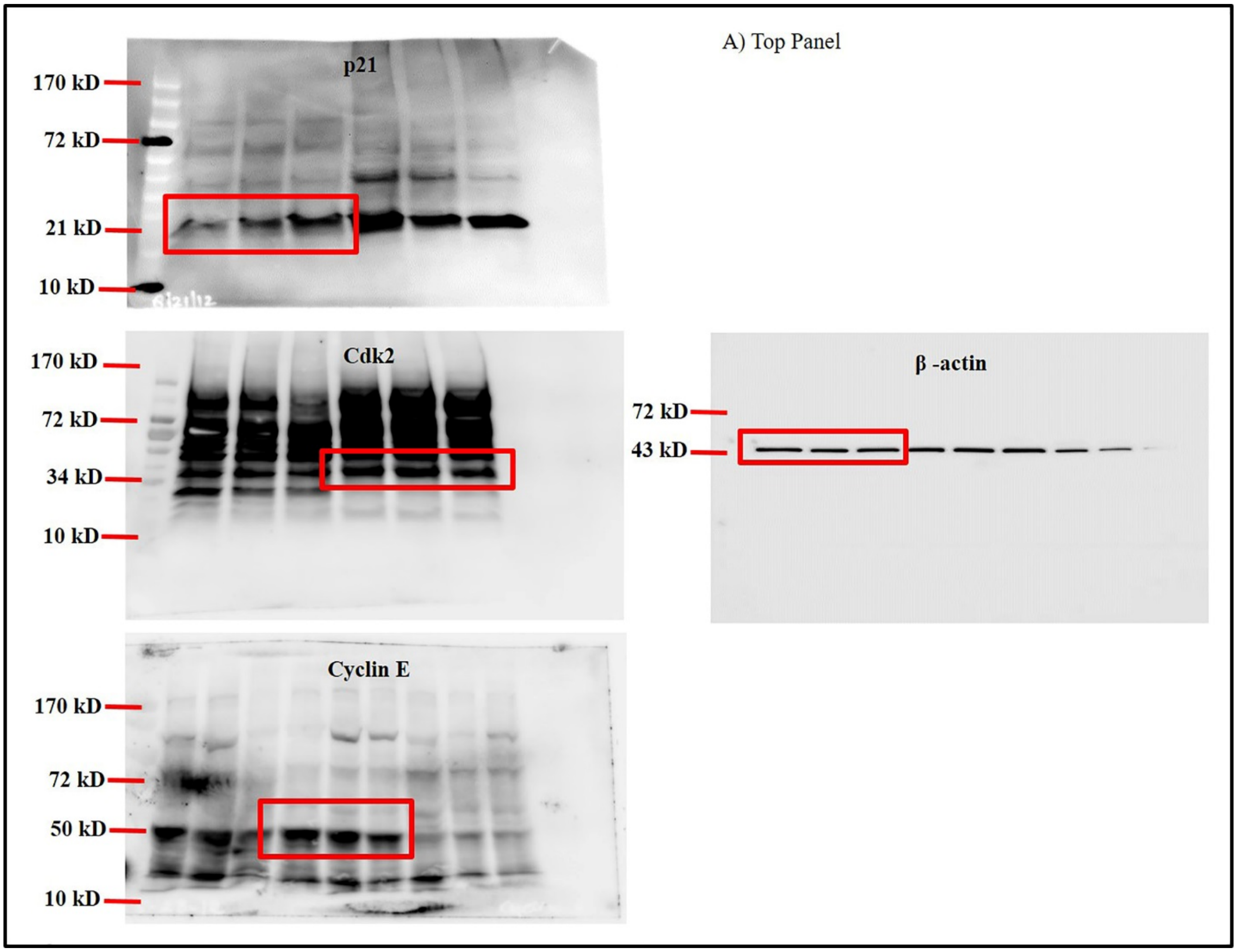

<u>Figure S3A:</u> Full western blots for proteins shown in figure 3 A top panel (p21, Cdk2, Cyclin E and β -actin).

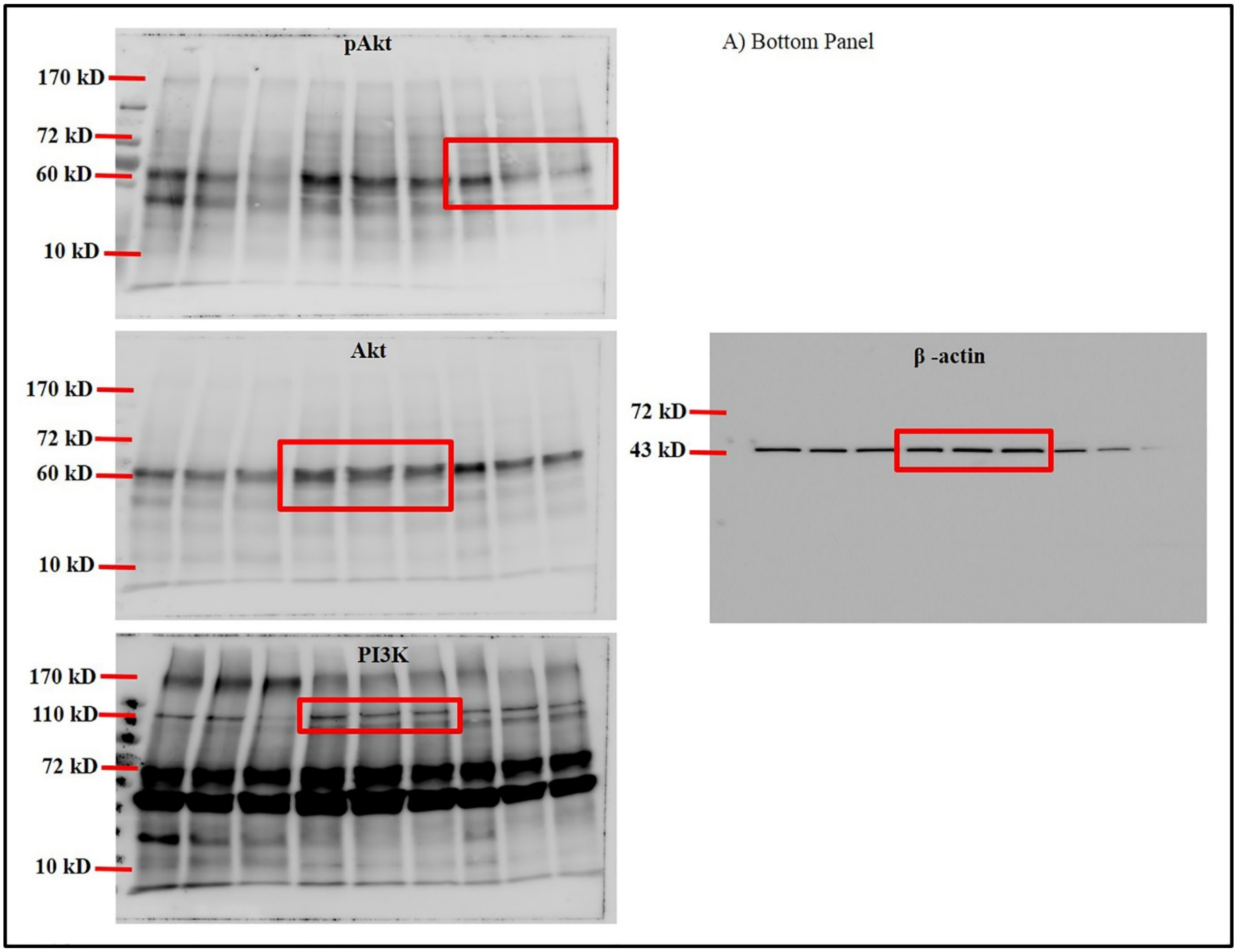

Figure S3A: **Full western blots for proteins shown in figure 3 A bottom panel (pAkt, Akt, PI3K and β -actin).**

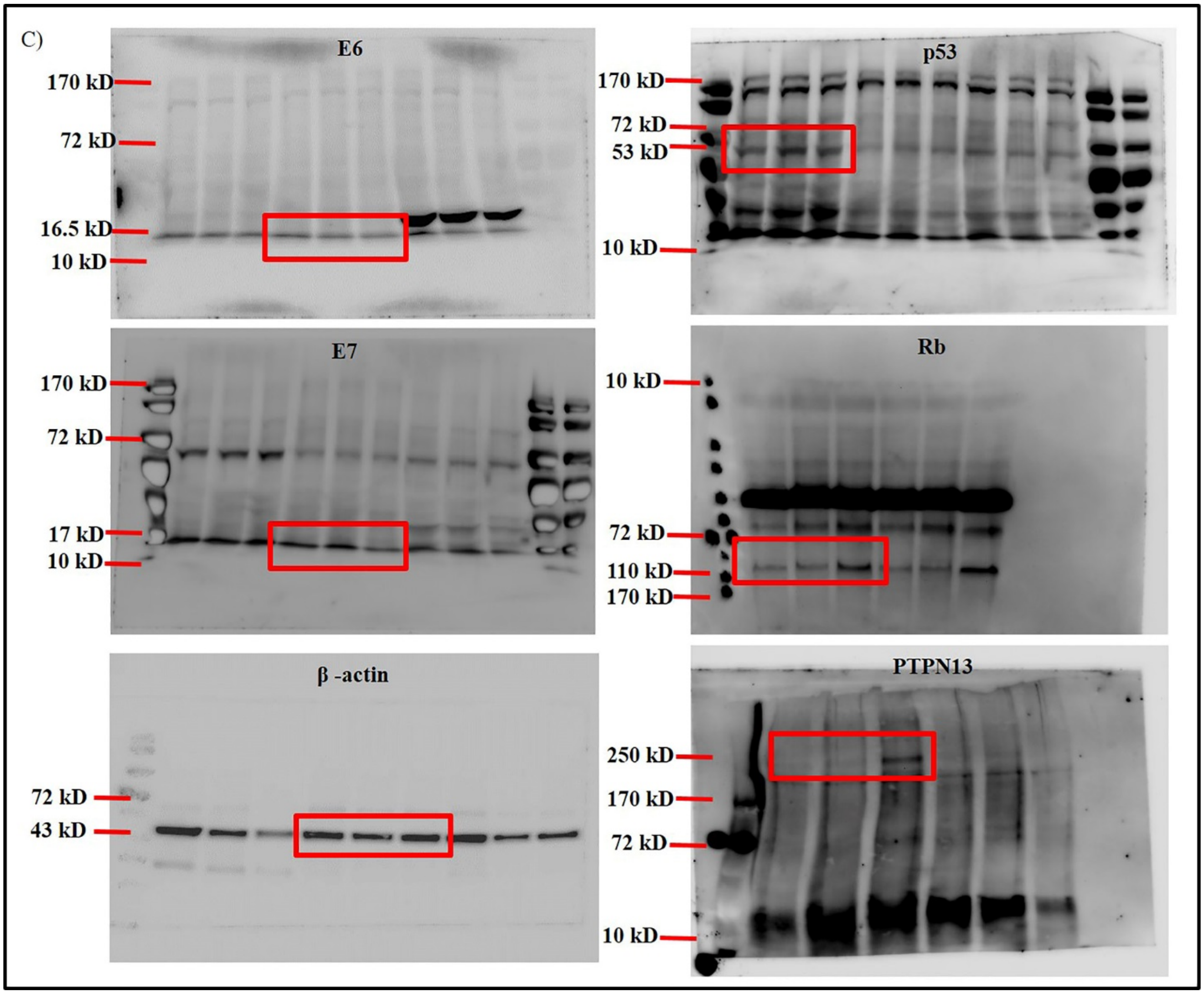

<u>Figure S4C:</u> Full western blots for proteins shown in figure 4 C (E6/ E7, p53, Rb, PTPN13 and β -actin), due to time constrain p53 and E7 were probed together in the same blot/membrane. Both proteins are far apart at the molecular weights and detected by anti-mouse secondary antibody.
